# Traumatic rupture of the diaphragm with pericardial diaphragmatic hernia

**DOI:** 10.1590/0100-3984.2017.0060

**Published:** 2018

**Authors:** Leonardo dos Santos Garcia, Alessandro Severo Alves de Melo, Luis Alcides Quevedo Cañete

**Affiliations:** 1 Complexo Hospitalar de Niterói (CHN), Niterói, RJ, Brazil.; 2 Hospital Universitário Antônio Pedro (HUAP), Niterói, RJ, Brazil.

*Dear Editor,*

**Figure 1:**
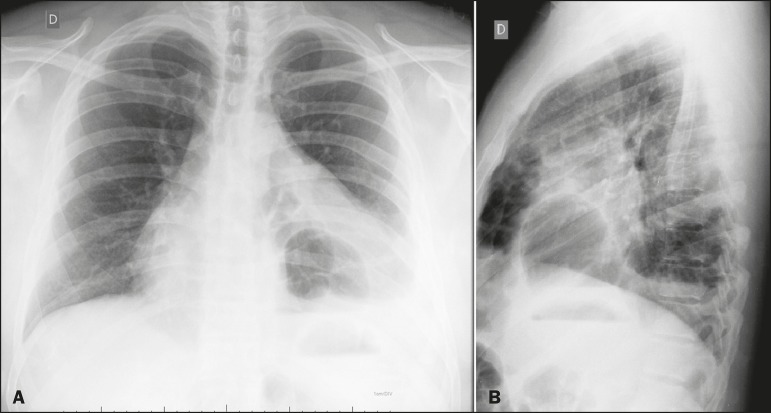
Posteroanterior and lateral X-rays of the chest (A and B, respectively) showing a gas-filled bowel loop projecting into the cardiac area.

**Figure 2:**
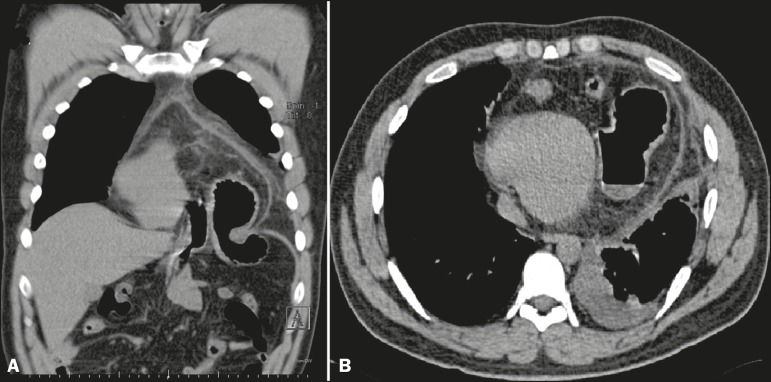
Computed tomography of the chest. Coronal reconstruction (A) and acquisition in the axial plane (B) demonstrating insinuation of the gastric antrum into the pericardial sac through the ring of a intrapericardial diaphragmatic hernia, together with pericardial thickening, cardiac compression, and small left pleural effusion.

A 28-year-old male patient was admitted to the hospital with a three-day history of respiratory difficulty (dyspnea) and chest pain radiating to the left shoulder. The patient had experienced thoracoabdominal trauma eight years prior, when he was hit by a car. He reported having noticed noises in the precordial region in the last six months, as well as discomfort in the epigastric region in the last month. On physical examination, auscultation revealed peristaltic noises in the precordial region, normal breath sounds on the right, and reduced breath sounds on the left. A chest X-ray showed gas-filled bowel loops projecting into the cardiac area (Figure 1). Computed tomography of the chest revealed a large anterior midline intrapericardial diaphragmatic hernia, with insinuation of the gastric antrum and a segment of the transverse colon into the pericardial sac, together with pericardial thickening, small left pleural effusion, and cardiac compression (Figure 2).

Diaphragmatic rupture is an uncommon condition caused by severe thoracoabdominal trauma^(1,2)^. Intrapericardial diaphragmatic hernia is even rarer, accounting for only 0.2-3.3% of all cases of diaphragmatic rupture^(3)^. It creates a direct communication between the pericardium and the peritoneal cavity, through a defect in the diaphragm. It can be congenital, due to failure of the transverse septum to develop, or can be associated with diaphragmatic rupture after trauma^(4)^.

The symptoms of intrapericardial diaphragmatic hernia vary depending on the location and organ involved. In the gastrointestinal tract, its presentation ranges from mild abdominal discomfort to acute abdomen with obstruction. When there is herniation of abdominal contents to the chest, there may be respiratory discomfort. In rare cases of intrapericardial diaphragmatic hernia, patients present with palpitations, chest discomfort, cardiac tamponade, and cardiogenic shock. Digestive, respiratory and cardiac symptoms may present alone or in combination^(1,5)^.

Traumatic lesions of the diaphragm are difficult to diagnose at their initial occurrence, because their clinical and radiological aspects are nonspecific, especially when the abdominal contents have not yet herniated^(1,2,6)^. When rupture is not initially recognized, the patient may remain asymptomatic or only mildly symptomatic for a long period of time (months or years), gradually evolving to herniation of the abdominal contents into the thoracic cavity^(2)^.

Currently, multidetector computed tomography is the method of choice for the diagnosis of diaphragmatic rupture, with a sensitivity and specificity of 61-87% and 72-100%, respectively, multiplanar reconstructions being of great value in establishing the diagnosis^(7)^. There are signs that indicate diaphragmatic hernia, and patients with a history of severe thoracoabdominal trauma should be screened for those signs through computed tomography of the chest^(7)^. Such signs include diaphragmatic discontinuity, focal constriction of the affected portion of the intestinal loop, constriction of the stomach at the site of herniation (collar sign), visualization of the nasogastric tube at the base of the affected hemithorax, and intrathoracic herniation of the abdominal contents into the thoracic cavity, the last being the most reliable sign.

Like computed tomography, magnetic resonance imaging demonstrates abrupt discontinuity of the diaphragm. However, the advantages of magnetic resonance imaging do not overcome its limitations in the management of trauma patients, making it most useful as an auxiliary imaging method^(8)^. Although ultrasound can reveal diaphragmatic ruptures, with a high positive predictive value, it also has limitations and is therefore not recommended as the primary method of evaluating such lesions^(8)^.
